# Seasonal Variations of the Nebraska Salt Marsh Microbiome: Environmental Impact, Antibiotic Resistance, and Unique Species

**DOI:** 10.3390/microorganisms13102369

**Published:** 2025-10-15

**Authors:** Emma K. Stock, Ketlyn Rota, Brandi Dunn, Madelynn Vasquez, Daniela Hernandez-Velazquez, Alyssia Lespes, Solenn Bosmans, Jace C. Smith, John A. Kyndt

**Affiliations:** 1 College of Science and Technology, Bellevue University, Bellevue, NE 68005, USA; emma.stock@evergreen.edu (E.K.S.); kdrota@my365.bellevue.edu (K.R.); bddunn@my365.bellevue.edu (B.D.); mvasquez4@huskers.unl.edu (M.V.); mrmerk971@gmail.com (J.C.S.); 2 Faculty of Biology, Universidad Veracruzana, Xalapa 91000, Mexico; danielandezv@hotmail.com; 3 Department GDT, Erasmus Brussels University of Applied Sciences and Art, 1090 Jette, Belgium; alyssia.lespes@student.ehb.be (A.L.); solenn.bosmans@student.ehb.be (S.B.)

**Keywords:** Nebraska salt marshes, purple bacteria, *Rhodobacter*, *Vibrio cholerae*, antibiotic resistance, seasonal microbiome variations

## Abstract

The Nebraska Salt Marshes are unique inland saltwater ecosystems, and this exploratory study is aimed at understanding the microbial composition and diversity that is providing the underlying support for these ecosystems. The microbiome shows both temporal and spatial variations that are concurrent with seasonal variations in salinity, temperature, and vegetation growth. Whole genome metagenomics analysis showed the predominance of purple non-sulfur bacteria in each season, indicating their importance in the marsh ecosystem. The fall season showed the highest microbial diversity and coincided with the highest levels of antimicrobial resistance markers to a variety of natural and synthetic antibiotics. In addition to the metagenomics approach, we also isolated and sequenced several unique species, most of them belonging to what appear to be new species of purple non-sulfur or purple sulfur bacteria. Both the metagenomics analysis and isolated species indicate that the nitrogen and sulfur cycling is well balanced in these marshes by a high relative abundance of purple bacteria. Noteworthy is the isolation of a new strain of *Vibrio cholerae*, which is a known human intestinal pathogen, that was predominantly present in the fall samples carrying several antibiotic resistance markers. Overall, the Nebraska salt marsh microbiome showcases both seasonal variations in microbial composition, a concerning prevalence of multiple antibiotic resistance, and the presence of unique bacterial species well-adapted to its distinctive alkaline and saline environment.

## 1. Introduction

Salt marshes on a global scale are vital ecosystems that provide numerous ecological services, such as water filtration, flood control, carbon sequestration, and habitat for diverse flora and fauna [1], and they play a vital role in coastal carbon cycling [2]. Unfortunately, around 50 percent of the world’s original salt marshes have been lost over the past century, and many of the remaining salt marshes worldwide suffer from serious degradation and loss of ecosystem function [3,4]. Although salt marshes in areas like the Mediterranean basin are well conserved and are even used to harvest salt for human consumption, there is a growing concern about their long-term sustainability, even for those locations. Human activities such as intensive agriculture and increased flooding patterns cause changes in soil salinity and vegetation [5,6]. A 2024 analysis by the Marine Conservation Institute found that many Mediterranean Marine Protected Areas (MPAs) and Natura 2000 sites, which contain salt marshes, lack effective management and enforcement to meet conservation goals [7]. According to the WWF’s 2019 study, only 2.48% of Mediterranean MPAs had a management plan, and 1.27% effectively implemented said plans. Even in countries with a higher number of MPAs, such as Spain and Croatia, MPAs tend to lack planning and enforcement for conservation goals. On a global scale, the major factors impacting salt marsh degradation and loss are coastal development and urbanization, pollution from fertilizer and sewage runoff, and sea level rise or changes in storm patterns that cause flooding or greater erosion [2,3,4,5,6].

The Nebraska salt marshes, located in and around Lincoln, Nebraska, are unique wetland ecosystems characterized by their saline soils and groundwater [8,9,10,11,12,13]. They are unique in the fact that they are not coastal marshes and are one of the few locations in the US where the naturally occurring groundwater is saline. These conditions provide habitat for several native plant and insect species that have adapted to grow in these distinct environments [8,9,10,11,12,13]. Overall, these marshes contribute to regional biodiversity and play a crucial role in maintaining ecological balance in the local environment.

One of the key aspects of wetlands that has garnered significant scientific interest is their microbiome—the community of microorganisms, including bacteria, fungi, and archaea, that inhabit these ecosystems [14,15]. The microbiome of a wetland is integral to its functionality, driving essential processes such as nutrient cycling, organic matter decomposition, and water quality maintenance. These activities subsequently support plant growth and sustain wetland health and productivity [14,15]. Studying the microbiome of wetlands, particularly in salt marshes, is important for several reasons. Although the diversity and functioning of microbial communities in these systems is highly underexplored, it is generally well-understood that the microbes in these wetlands are the key actors of the main biogeochemical cycles (mainly N, C and Fe-S), and many biochemical conversions catalyzed by microbes can ultimately control vegetation growth in wetlands. Therefore, any dysbiosis in these microbial communities can result in disruption of these nutrient cycles with larger environmental consequences to these ecosystems [14,16,17,18]. In addition, these microorganisms are highly responsive to environmental changes, making them excellent indicators of ecosystem health and stability [19]. Therefore, understanding the dynamics of microbial communities can provide insights into the resilience of wetlands to environmental stressors, such as climate change, pollution, and land-use changes [19]. This is particularly relevant for the Nebraska salt marshes, which experience seasonal variations in temperature, precipitation, and salinity, while also facing increasing pressure from nearby urban development. Urban expansion and consequential changes to the hydrological systems have endangered the continuing existence of these unique salt marshes. Only about 4000 acres remain scattered throughout the region of the estimated 20,000 acres that once existed [13]. In particular, the Marsh Wren saline wetland at the Salt Creek valley, near Lincoln, NE, is the most endangered by growing urban development and has been used for protective restoration, mainly focused on the manipulation of hydrology through pumping of saline groundwater [20].

We previously reported a singular study on the Nebraska salt marsh microbiome at the Marsh Wren restoration area (near Lincoln, NE, USA), which provided a snapshot of the diversity and complexity of the microbial life in this unique ecosystem [15]. Another 16S rRNA-based sequencing study previously investigated the microbial communities in the more western alkaline Nebraska Sandhill lakes [21]. Although these studies provided interesting insights and a good baseline of what this unique microbiome looks like at some of the locations, they were only single time point snapshots and used 16S metagenomic analysis instead of whole genome-based metagenomics. The latter could provide more in-depth information at the species level, as well as the potential presence of antimicrobial or virulence markers.

By investigating how the microbiome of the Nebraska salt marshes fluctuates over the seasons, we can gain a deeper understanding of the ecological processes underpinning these wetlands. Identifying microbial species that are consistently present or fluctuate in certain seasons is important to understand for future conservation studies. Seasonal changes can influence the microbial diversity and function, affecting the overall health and sustainability of the marshes. This knowledge can inform conservation efforts, guide the management of wetland resources, and contribute to the broader field of microbial ecology. To address these gaps, we conducted year-round sampling (over a 16-month period) across multiple Nebraska salt marsh locations at the Marsh Wren wetland restoration area, to investigate both temporal and spatial patterns of microbial diversity. Understanding how the microbiome adapts to the extreme weather change of the Midwest seasons could help with the preservation of the salt marshes. A large quantity of decomposing plants could for example cause an increase in gases like carbon and nitrogen if there is a microbial dysbiosis that limits the nutrient recycling. This study can enhance the understanding of the bacteria that are present and their interactions in a natural environment. This will potentially allow for the management and optimization of bacteria in these and other natural bodies of saltwater. The Nebraska salt marshes are remnants of prehistoric oceans, and preserving these wetlands is not only important for the ecosystem of the marsh but also allows us to obtain a historical glimpse of the evolution and adaptation of species to more extreme environments.

## 2. Materials and Methods

### 2.1. Sample Collection

Water samples were collected from two locations at the ponds within the Nebraska salt marshes near Lincoln, Nebraska (Location 1: Lat 40°52′54.42″ N; Lon. 96°39′35.82″ W, and Location 2: 40°52′58.7634″ N; 96°39′22.248″ W). Sampling sites were at the edge of the waterline and less than 1 m deep. Sampling sites were selected at the halfway point of the longitudinal side of the ponds and taken along the entire depth. For the December samples, a thin (about 1 cm) ice layer had to be broken to access the liquid water underneath. The salinity was measured with a handheld salinity hydrometer (Fluvial Sea, Montreal, QC, Canada), while the nitrate, nitrite, and pH values were measured with 5-in-1 EasyStrips (Tetra, Spectrum Brands, VA, USA). Samples were collected in duplicate (for October, December and April) or triplicate (for March and July) at each location and processed individually. Each site was sampled using 50 mL sterile collection tubes and immediately transferred to the lab where they were stored at 4 °C the same day. The next day, the samples were processed for DNA extraction. Sampling was performed on 27 March 2024, 10 July 2024, 17 October 2024, 12 December 2024, 15 April 2025, and 31 July 2025, at both locations, except for the first sampling (March 2024) which was only completed at location 1. The last July sampling (2025) was only performed at location 1 because there was excessive flooding of location 2 due to rainstorms, which limited access to the exact sampling site.

### 2.2. Cultivation

RCVB agar plates [22] were prepared with the pH adjusted to 9.5 and supplemented with 10 g/L NaCl. After autoclaving, the medium was enriched with niacin (1 mg/L), thiamine (1 mg/L), and vitamin B12 (10 mg/L) under sterile conditions. To isolate bacterial cultures, 100 μL of the collected water samples was spread onto the prepared plates. Incubation was carried out at 25 °C under both aerobic and anaerobic conditions, in light and dark chambers, to promote bacterial growth. After a two-week period, distinct colonies were selected and sub-cultured twice on fresh plates to obtain pure isolates for further analysis.

### 2.3. DNA Extraction

In addition to culturing bacteria, 6 mL water samples were subjected to centrifugation to concentrate microbial cells. Samples were spun at max speed (20,000× *g*) for 15 min. The resulting pellets, containing the microbial biomass, were carefully collected and stored at −20 °C until further processing.

DNA extraction was performed on both the cultured microbial colonies and the centrifuged water pellets. For the agar plate colonies, individual colonies were picked and subjected to DNA extraction; for the water samples, DNA was extracted directly from the pellets. DNA extraction was performed using the PureLink Microbiome kit (Invitrogen, Waltham, MA, USA). DNA analysis using Qubit and NanoDrop showed final concentrations ranging from 10.6 ng/µL to 134 ng/µL and A260/280 ratios ranging from 1.6 to 1.9. For whole genome-based sequencing, we used 100–500 ng of each sample.

### 2.4. High Throughput WGS Shotgun Sequencing

Whole genome metagenomics sequencing libraries were prepared using the Illumina DNA Library Prep kit, and the library quality and concentration were assessed using Qubit. Libraries were sequenced by an Illumina MiniSeq using 500 μL of a 1.8 pM library using High-output cassettes. Paired-end (2 × 150 bp) sequencing generated between 671,256 and 4,344,468 reads (>Q30) and 102–656 Mbps of data for each of the samples. One replica sample (from both locations of the July 2024 samplings) failed to produce sufficient DNA for sequencing, and only single samples were used from those timepoints.

### 2.5. WGS Metagenomic Analysis

Quality control of the raw reads was performed using FASTQC in BaseSpace (Illumina; version 1.0.0). Adapter sequences were removed, and reads with low quality scores (average score < 20) were filtered out using the FASTQ Toolkit within BaseSpace (Illumina, version 2.2.0). Taxonomic classification was analyzed using the BV-BRC Taxonomic Classification pipeline [23]. The WGS pipelines are described in Lu et al., 2022 [24]. To remove host contamination, HISAT2 (v2.2.1) was used to map the reads against the *Homo sapiens* reference genome. Reads identified as host-derived were then discarded from the dataset. Then, the host-removed reads were used in the Kraken2 command [25]. The Microbiome Analysis was used, which is an end-to-end pipeline that uses Kraken2 to identify taxa at the species level. This pipeline uses Bracken (v2.7), which is a companion program to Kraken2 and the other tools in the Kraken suite. Bracken uses Kraken’s output to estimate the abundance of those taxonomic units, particularly at the species level. Kraken 2 assigns taxonomic labels to metagenomic DNA sequences using the exact alignment of k-mers. The BV-BRC Database was used, which is the Kraken2 database that includes the RefSeq complete genomes and protein/nucleotide sequences for the following: Archaea, Bacteria, Plasmid, Viral, Human GRCh38, Fungi, Plant, Protozoa, and UniVec (an NCBI-supplied database of vector, adapter, linker, and primer sequences that may be contaminating sequencing projects and/or assemblies). The default confidence interval was 0.1. Both KRONA and Sankey Diagrams were generated as output from the BV-BRC Taxonomic Classification pipeline. The output MultiQC (v1.11) sample data were uploaded to ClustVis (v1.0) for multicomponent PCA analysis and multidimensional visualization [26]. In ClustVis, unit variance scaling was applied to the annotated datasets, and SVD (singular value decomposition) with imputation was used to calculate the principal components in the PCA analyses.

The MultiQC report summarized the identification results from multiple samples, to identify which microbes were unique within each sample or common amongst all samples. The values were provided as the robust z-score calculated from the Kraken2 report. The robust z-score is the median absolute deviation, which normalizes the data against a control group to determine whether a taxon’s abundance is significantly different than expected, making it a measure of relative differential abundance. This method is chosen to reduce the impact from outliers in the data, providing a more reliable measure of relative position within the data distribution. A positive robust z-score indicates that the number of fragments assigned to those taxa was above the median or central tendency of the data. Conversely, a negative robust z-score indicates that the number of fragments assigned to those taxa was below the median or central tendency of the data. The magnitude (represented as the value of the z-score) indicates the distance of the data point from the central tendency in terms of the robust measure of dispersion.

Alpha-diversity is measured as the observed richness (number of taxa) or evenness (the relative abundances of those taxa) of an average sample within one sample [27]. These were calculated with the KrakenTools alpha_diversity.py script. Beta-diversity, defined as the variability in community composition (the identity of taxa observed) between samples within a group [27], was calculated using the KrakenTools beta_diversity.py script to generate a Bray–Curtis dissimilarity matrix for pairwise comparisons among three microbiome samples. A value of 0 indicates that two samples are identical, whereas a value of 1 reflects maximal divergence [28].

Linear discriminant analysis Effect Size (LEfSe) analysis is a bioinformatics method that uses Linear Discriminant Analysis (LDA) and the effect size to discover and explain biological and statistical differences between microbial organisms between two or more groups. It first performs non-parametric Kruskal–Wallis tests and then uses LDA to measure the size and consistency of these differences [29]. LEfSE software was obtained from Github (https://github.com/SegataLab/lefse, accessed on 5 October 2025) and ran in Python (v3.13) with the following parameters: Kruskal–Wallis alpha (class level): 0.05; Wilcoxen alpha (pairwise within subclass): 0.05; LDA score threshold (absolute): 2.0; multiclass strategy: all-against-all; bootstraps: 50; normalization during formatting: sum-scaling to 1,000,000 (LEfSe default via -o 1000000); flags: -a 0.05 -w 0.05 -l 2.0 -b 50 -t all-against-all (with the input prepared using -c 1 -s 2 -u 3 -o 1000000).

For antimicrobial resistance analyses, we used the Metagenomic Read Mapping Service in BV-BRC, which uses KMA (k-mer alignment) [30], to align reads against antibiotic resistance genes of the CARD database [31]. KMA maps raw reads directly against these databases and uses k-mer seeding to speed up mapping and the Needleman–Wunsch algorithm to accurately align extensions from k-mer seeds.

### 2.6. WGS Assembly and Analysis

For the WGS of the isolated species, quality filtration (>Q30) and adapter trimming were performed by the MiniSeq software (v.2.2.1), and quality control of the reads was performed using FASTQC (version 1.0.0) within BaseSpace (Illumina), using a k-mer size of 5 and contamination filtering. The genome was assembled de novo through BV-BRC [23] using Unicycler (v0.4.8) [32]. The final genomes were 98–100% complete, with no significant contamination (0–2%) according to CheckM (v1.1.6) [33], and were annotated by RASTtk [34]. Default parameters were used for all software applications unless otherwise noted.

The ‘Similar Genome Finder’ tool in BV-BRC was used to identify the closest relatives of each genome, which uses Mash/MinHash [35]. Mash reduces large sequences and sequence-sets to small representative sketches, from which global mutation distances can be rapidly estimated. The MinHash dimensionality-reduction technique includes a pairwise mutation distance and *p* value significance test, enabling the efficient clustering and search of massive sequence collections.

For average nucleotide identity (ANI) analysis, we used JSpecies, which uses a pairwise genome comparison algorithm to measure the probability of genomes belonging to the same species, with an arbitrary species cutoff of 95% [36].

## 3. Results

### 3.1. Seasonal Microbial Variations

#### 3.1.1. Overall Comparison

**Figure 1:**
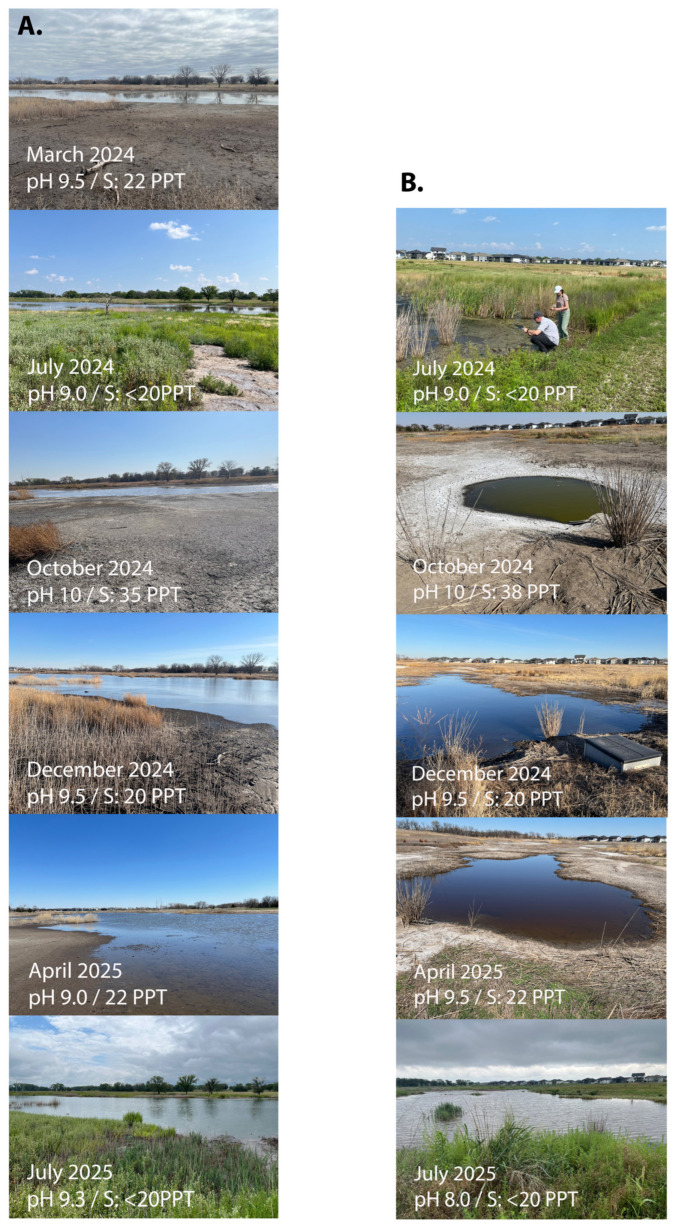
Seasonal images from the sampling sites at location 1 (**A**) and at location 2 (**B**) at the Nebraska salt marshes. Each image has the month and on-site measured pH and salinity (S) included.

Over a period of 16 months (March 2024–July 2025), twenty-three water samples were taken from two locations at the Nebraska salt marshes and analyzed for their nitrate/nitrite, pH, salinity, and microbial composition (Figure 1). Location 1 is the major body of water at the Lincoln salt marshes, while location 2 is one of the smaller ponds (located about 400 m away), which is more susceptible to overflooding and seasonal desiccation. Both locations are within the Marsh Wren saline wetland restoration project area. The nitrate/nitrite concentrations for all samples were within safe acceptable ranges (nitrate 0–5 ppm, nitrite 0–0.2 ppm), and the pH of each location and date showed little variation (pH 9–10). Surprisingly, there was substantial variation in the salt concentration in the various samples (Figure 1). The fall sample (in October 2024) showed the highest salinity with 35 and 38 PPT for each location, while all the other seasonal samples were lower, with the lowest salinity observed in the July samples (less than 20 PPT). We had previously sampled at location 1 for an earlier baseline study (in early October 2020, [15]) and had observed high salinity values (>38 PPT), which is consistent with the current fall measurements. The lower salinity in most of the other samples was somewhat surprising given the earlier reports on the history and nature of the salt marshes. However, as can be seen in Figure 1, the October sampling sites also showed the most drought and had retracted water lines when compared to the other seasons, which undoubtedly increased the osmotic solutes in the pond water. In contrast, the summer samplings (July) showed the lowest salinity and also a higher degree of vegetation growth around the ponds (Figure 1). This is likely due to increased rainfall and early summer storms that are common in Nebraska and is consistent with the fact that the highest level of precipitation in Lincoln, Nebraska, in 2024 was in July, with 7.23 inches (reported by the National Weather Service NOAA). The apparent seasonal variation in the salt levels of these salt marshes will likely have an impact on microbiome changes over the seasons.

**Figure 2:**
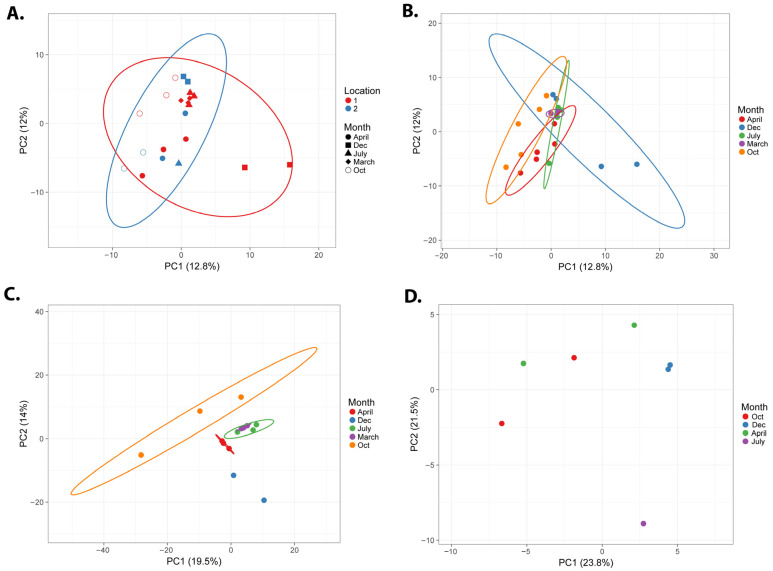
PCA analysis of the seasonal microbiome samples of the Nebraska salt marshes. Unit variance scaling was applied to the annotated datasets, and SVD with imputation was used to calculate the principal components. The X and Y axes show the principal component 1 and principal component 2 with the percentage of total variance indicated on the axes. Prediction ellipses are such that, with a probability of 0.95, a new observation of the same group will fall inside the ellipse. (**A**) PCA analysis of all samples with the locations grouped, and (**B**) is the same with the months grouped (and location ignored), *n* = 21 data points. (**C**) shows the PCA analysis for location 1 (*n* = 14), and (**D**) is the PCA for location 2 only (*n* = 7).

We analyzed the microbial composition of all of the seasonal and spatially different samples by high-throughput whole genome metagenomics. A multidimensional PCA analysis of the seasonal microbiome samples (Figure 2) shows that samples from the two locations are distinct from each other (Figure 2A red and blue ellipses, probability > 0.95), while the location 1 samples show more variety than the location 2 samples. When only seasonal variation is considered (Figure 2B), there is seasonal overlap between the samples; however, the samples from October and December show the highest degree of variation (Figure 2B, orange and blue ellipses). Given the different variability between the two locations, we analyzed each location separately for their alpha- and beta-diversity and microbial composition.

**Figure 3:**
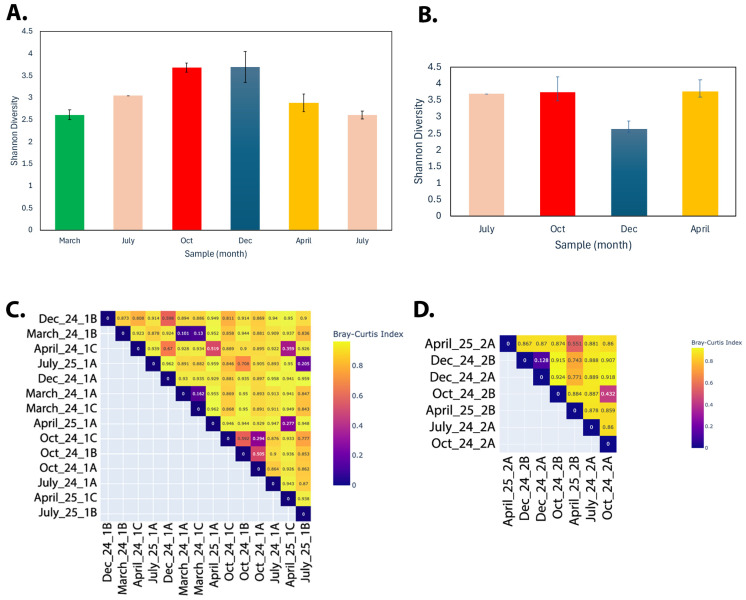
(**A**,**B**) Average Shannon Index alpha-diversity for location 1 (**A**) and location 2 (**B**) samples. A higher value indicates a larger number of species and evenness of their abundance. (**C**,**D**) Heatmaps of beta-diversity for location 1 (**C**) and location 2 (**D**) samples. Beta-diversity is calculated using the Bray–Curtis Index of dissimilarity. If two samples have the same microbes in the same abundance, the dissimilarity is 0. If there are no shared microbes between them, the dissimilarity is 1.

The alpha-diversity for each sample was calculated as the Shannon diversity, Simpson diversity, Simpson reciprocal index, Berger–Parker index, and Fisher’s index (overview in Supplemental Table S1). All alpha-diversity index calculations showed a similar pattern, and Figure 3A,B are graphical illustrations of the average Shannon diversity index for the different sampling months. For location 1, the March samples showed the lowest diversity, while the October and December samples had the highest diversity. For location 2, October and April had the highest and December the lowest diversity (although note that location 2 did not have a March location sampling).

The beta-diversity heatmaps (Figure 3C,D) showed strong similarity among the replica samples (as expected), while the seasonal samples differed significantly in microbial abundance, reflected by a higher level of dissimilarity in the Bray–Curtis index.

#### 3.1.2. Microbial Composition Analysis

##### Location 1

A PCA plot of the location 1 samples showed the highest variation in the October samples (Figure 2C), although the samples generally clustered by season. It is noteworthy that the microbiome samples collected in the same season strongly aligned over the two years. For example, the July 2025 and July 2024 samples and the March 2024 and April 2025 samples grouped closely in the multidimensional analyses. This indicates some level of resilience and consistency of the microbiome over the two-year study period.

**Figure 4:**
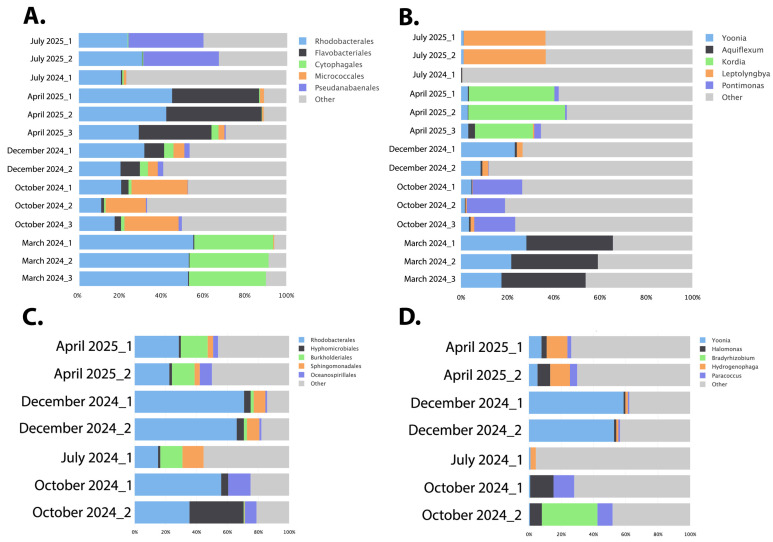
Bracken overview of the top taxa at location 1 (**A**,**B**) and location 2 (**C**,**D**). Figures (**A**,**C**) show the top taxa at the Order level, while figures (**B**,**D**) show the top taxa at the genus level for each sample. The bottom axis provides the number of fragments as a percentage of the total.

A Bracken overview of the top taxa at the Order level in each sample (Figure 4A) illustrates that *Rhodobacterales* was one of the top taxa in each of the seasonal samples (light blue bars in Figure 4A). The largest differentiating taxa were *Cytophagales* for the March samples, *Micrococcales* for the October samples, *Flavobacteriales* for the April samples, and *Pseudanabaenales* for the July samples. Interestingly, cyanobacteria (*Pseudanabaenales* and *Synechococcales*) were only found in high abundance in the July samples.

At the genus level (Figure 4B), the highest March representation was *Aquiflexum* and *Yoonia*, while it was *Pontimonas* and *Yoonia* in October, *Yoonia* in December, *Kordia* in April, and the cyanobacteria *Leptolyngbya* in the July samples.

##### Location 2

Although the location 2 sampling had a lower number of samples collected, a similar pattern of microbial variation to location 1 can be seen with the PCA plot (Figure 2D). Each of the sampled microbiomes generally separates well based on their season of collection. At location 2, the July sample appears to be more divergent from the other samples in the PCA analysis. A Bracken analysis of the top taxa at the Order level shows consistently that *Rhodobacterales* are highly represented in each season in the Nebraska salt marshes (Figure 4C). Both the July and April samples had a high representation of *Burkholderiales*, but the April samples also showed a relatively high representation of *Sphingomonadales* and *Oceanospirillales*. The October samples showed particularly high levels of *Hyphomicrobiales*. With the exception of *Rhodobacterales*, the top taxa from each seasonal sample were different between the location 1 and location 2 samplings, which is consistent with the observed distinction of the two locations in the overall PCA analysis (Figure 2A). In location 2, there is a notable absence of cyanobacteria or algae in the top represented taxa at any of the samples.

At the genus level, the October collections showed high levels of *Halomonas* and *Paracoccus*, while one replicate also exhibited elevated *Bradyrhizobium* (Figure 4D). The latter are common nitrogen-fixing soil bacteria that often form symbiotic relationships with plants, suggesting that some plant root material may have been present in that water collection. As in location 1, the December dataset contained high levels of *Yoonia*. The April and July samples were more diverse, but *Hydrogenophaga* was highly represented in both months.

**Figure 5:**
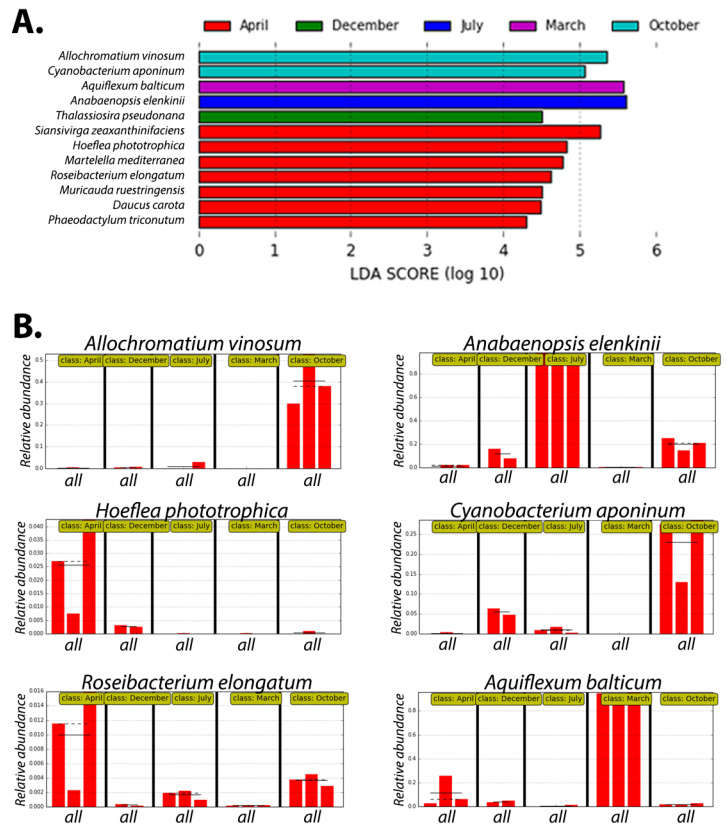
LEfSE analysis for the seasonal microbiome samples for location 1 showing the statistically different species for each temporal sample (**A**). Species names are on the Y axis, and the logarithmic LDA score for each is on the X axis. The colors represent the sampling month. (**B**) LEfSE feature plots of selected species showing the relative abundance of each species on the Y axis and sampling time indicated as ‘class’ on top of each graph.

The abovementioned PCA analyses (Figure 2) and relative abundance plots (Figure 4) provide a good high-level overview of the microbial variation between the seasonal samples. However, to specifically identify the main differentiating species between the temporal datasets, we performed an additional LDA analysis. PCA aims to find the directions of maximum variance in the data, while LDA aims to find the projection that best separates the classes in the data. Figure 5 shows the results of the LEfSE analysis, which is specifically designed for LDA analysis on microbiome datasets [29]. All of the differentiating species identified (Figure 5) are isolated from marine or halophilic environments, and the majority of them are photosynthetic species belonging to either purple non-sulfur, purple sulfur, or cyanobacteria.

*Allochromatium vinosum* is one of the highest differentiating factors in the October samples (Figure 5A,B) and is a purple sulfur bacterium belonging to the *Chromatiaceae* [37]. *A. vinosum* plays a significant role in nutrient cycling by reoxidizing sulfide produced by other bacteria in anoxic layers. Purple sulfur bacteria in general are major players in the reoxidation of sulfide in both freshwater and marine environments [15,37,38]. The photosynthetic bacteria found in the April samples, *Hoeflea phototrophica* and *Roseibacter elongatum*, belong to a specific group of aerobic anoxygenic phototrophic (AAP) purple bacteria, which are obligate aerobic (as opposed to other anoxygenic purple bacteria that can perform anaerobic photosynthesis). AAP bacteria are thought to play an important role in carbon cycling by relying on organic matter and acting as sinks for dissolved organic carbon. There is still a knowledge gap in research areas regarding the evolution and ecology of AAPs; however, their photosynthetic machinery and genomic makeup is most related to purple non-sulfur bacteria [39,40]. The high abundance of *Rhodobacterales* in the seasonal samples above (Figure 4) is consistent with the differential occurrence of these AAP/PNS species.

The oxygenic phototrophs, cyanobacteria and diatoms, are found differentially in October, July, and April, with *Anabaenopsis elenkinii* (Figure 5A,B) specific for July and *Cyanobacterium aponicum* for October (Figure 5A,B). The identification of *Anabaenopsis* in the July samples is not too surprising, given the higher relative abundance of cyanobacteria we noted in this sample earlier (Figure 4A). *Cyanobacterium aponicum* is a halophilic cyanobacterium that was shown to tolerate high salt concentrations and temperatures up to 45 °C [41,42], which correlates well with the fact that October had the highest salinity of our samples (Figure 1).

*Aquiflexum balticum* (found in March LDA samples, Figure 5) is a marine heterotrophic bacterial species that was originally isolated from the Baltic Sea in Finland and typically contributes to the degradation of plankton blooms in an aquatic environment [43]. *Aquiflexum* was also found with high relative abundance in the March sample in Figure 4 above. The LDA April samples contained two marine heterotrophic Flavobacterial species, *Siansivirga zeaxanthinifaciens*, isolated from China Sea in Taiwan [44], and *Muricauda ruestringensis*, isolated from the North Sea [45]. This correlates well with the higher abundance of *Flavobacteriales* for the April samples in Figure 4A above. *Martelella mediterranea* is a halotolerant aerobic species isolated from a subterranean saline lake in Spain [46], and although its nitrogen-fixing capabilities have not been tested, it belongs to the *Rhizobiaceae*, which contains many nitrogen-fixing microbes.

Overall, the observations from the relative abundance variations correlate well with the results from the LDA analysis, given that many of the high abundance bacterial families or species were found to differentially vary by season in the LDA analysis.

#### 3.1.3. Antibiotic Resistance Analysis

The presence of antibiotic resistance gene markers in environmental samples is of growing concern, because it can contribute to the spread of antibiotic resistance in potential pathogenic strains, contributing to the growing challenges of antibiotic resistance infections in healthcare. Therefore, we performed a search for potential antimicrobial resistance (AMR) markers in our seasonal samples of the Nebraska salt marshes.

A Metagenomic Read Mapping against the CARD database [30,47] was performed in BV-BRC for each of the samples. A total of 21 AMR targets were identified in the samples (see Supplemental Table S2); however, there was a substantial seasonal difference in the occurrence of these markers. In location 1, the majority of AMR genes were present in October (14 targets) and December (9 targets), while the other months had substantially fewer (5 in March and only 1 in both July and April). For location 2, the April samples had the highest occurrence of AMR genes (eight targets), while the other months each had three–four.

Many of the hits were spectinomycin resistance markers that matched against the 16S templates of *E. coli*, *Salmonella*, and *Neisseria*, but there were several more specific resistance markers, for example against paromycin, linezolid, kanamycin, pulvomycin, macrolides, chloramphenicol, eflamycin, and clarithromycin. Interestingly, nearly all of the seasonal samples showed resistance markers against clindamycin, which is a lincosamide antibiotic medication that works by interfering with bacterial protein synthesis. It is a semisynthetic antibiotic that is used for the treatment of a number of bacterial infections, including osteomyelitis (bone) or joint infections, pelvic inflammatory disease, strep throat, pneumonia, acute otitis media, and endocarditis. It can also be used to treat MRSA infections. Clindamycin resistance in the environment and healthcare settings is of growing concern [48,49,50,51], and its widespread detection in environmental salt marsh samples highlights its contribution to this larger problem.

The occurrence of several AMR markers in a more excluded, high salt, and high pH aquatic environment that is mainly fed by groundwater and precipitation is certainly concerning and shows the widespread issue with antibiotic resistance. This is not limited to natural antibiotics but also includes semisynthetic and synthetic antibiotic groups. The seasonal variation, with high levels of AMR in October (location 1), December (location 1) and April (location 2), indicates that there are environmental influences that drive these seasonal microbial variations that deserve further study.

### 3.2. Isolated Species and Their Initial Characterization

Given the occurrence of *Rhodobacterales* in all samples and seasons and the fact that our earlier 16S rRNA-based metagenomics report [15] showed the presence of unique purple non-sulfur bacteria, we attempted to cultivate photosynthetic species on RCVB media, at pH 9.5 and supplemented with 10 g/L NaCl, with the prospect of the isolation and identification of some unique species. After both aerobic and anaerobic growth under either dark or light conditions we selected several yellow-, reddish-, or brown-colored bacterial colonies that were purified by repetitive plating and eventually used for genomic DNA extraction and whole genome sequencing.

Table 1 provides an overview of the isolated species and their whole genome characteristics. For each of these, we identified the closest genome relative, using the ‘Similar genome finder’ tool in BV-BRC, and calculated the average nucleotide identity (ANI) using JSpecies. It has been shown that bacteria with ANI > 95% can be recognized as belonging to the same species [36], while bacteria with ANI < 90% would be recognized in most cases as separate species. Those with values between 90 and 95% identity may be argued either way depending on other properties. With the exception of the *Oceanimonas*, *Pseudomonas*, and *Vibrio* species, all of the new genomes show ANI values well below the species ANI cutoff and could therefore potentially be recognized as new species, given further physiological and morphological analysis (Table 1).

**Figure 6:**
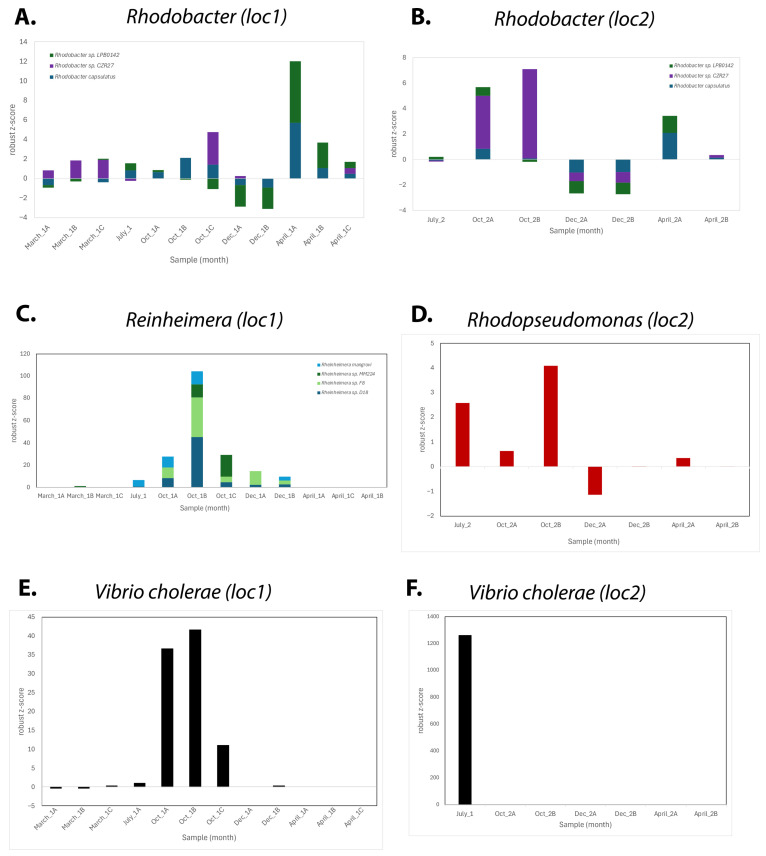
Relative abundance of selected species for each seasonal sampling over a 1-year period. The robust z-score is calculated from the from the Kraken2 report, which identified sequencing reads using taxonomic mapping in BV-BRC. (**A**,**B**) show *Rhodobacter* sp. at locations 1 and 2, respectively; (**C**) shows *Reinheimera* sp. presence at location 1; (**D**) shows *Rhodopseudomonas palustris* abundance at location 2; (**E**,**F**) show *Vibrio cholera* abundance at locations 1 and 2, respectively.

Three of the isolated and sequenced species belong to the *Rhodobacterales*: *Rhodobacter*, *Roseinatronobacter*, and *Paracoccus* (Table 1 orange rows). The *Rhodobacter* species was isolated from both location 1 and location 2, and *Rhodobacter* was found to be present in all of the metagenomic datasets. The metagenomics data showed the presence of three *Rhodobacter* species, and when plotting the normalized relative abundance of these for each location, we can see that, overall, they are most present in the October and April samples (Figure 6A,B). On the other hand, the presence of *Rhodobacter* falls below the mean average during the December months for both locations. The genome of the isolated species (designated *Rhodobacter* sp. NSM, for Nebraska Salt Marsh strain) shows the closest overall homology to *Rhodobacter megalophilus* DSM 18937, which was isolated from soil of the Indian Himalayas [52]. A JSpecies comparison of the average nucleotide identity showed only 84.4% ANI, indicating that strain NSM is likely a new uncharacterized species of *Rhodobacter*. The *Roseintronobacter* NSM species is even more distant from any relative, with only 75.8% ANI to *Roseinatronobacter thiooxidans* DSM 13087, which indicates that this may even be a separate new genus. Further cultivation and physiological studies are currently underway to characterize several of these new strains.

Three other species were identified as belonging to *Hyphomicrobiales*, including potentially new species of *Rhodopseudomonas*, *Salinarimonas*, and *Marinobacter* (Table 1, blue rows). *Rhodopseudomonas* was isolated from the location 2 samples, and the metagenomic seasonal analysis showed *Hyphomicrobiales* largely represented in the location 2 October samples (Figure 4C). The abundance plots of *Rhodopseudomonas* for location 2 show that it is most present in the October and July samples (Figure 6D). *Rhodopseudomonas* are purple non-sulfur bacteria that belong to *Nitrobacteriaceae*; many species play important environmental roles in nitrogen fixation and have been shown to function as plant growth promoting microbes [53,54,55,56,57,58,59]. The closest relative to the isolated NSM strain is *Rhodopseudomonas* sp. HaA2, which was isolated from a shallow rainwater pond in the Netherlands [60], and its genome was found to be significantly different from the type strain *Rhodopseudomonas palustris* DSM 123 [61]. With 90% ANI, further analysis will be needed to determine whether the strains NSM and HaA2 belong to a new separate species group.

Our main focus was on isolating photosynthetic purple non-sulfur bacteria; however, it is interesting that the species *Rheinheimera* was isolated from anaerobic grown RCV plates as the only purple sulfur *Chromatiaceae* species isolated. *Chromatiaceae* are one of the three families of purple sulfur bacteria, together with the *Ectothiorhodospiraceae* and *Halorhodospiraceae* families [37]. Purple sulfur bacteria play an essential role in the environmental sulfur metabolism. With an ANI of only 84.5% to the genome of the closest relative, *Rheinheimera* sp. YQF-2, the newly isolated strain is very likely a new species of the *Chromatiaceae* family and will be further characterized.

Another unexpected isolate was *Pseudomonas* sp. NSM, which belongs to the *Pseudomonadales*, and its closest relatives are *Pseudomonas oleovorans* and *Psd. mendocina*. With an ANI of 98.8%, this isolate is most likely a strain belonging to *Pseudomonas oleovorans* (recently renamed *Ectopseudomonas oleovorans*). When analyzing its genome, it became apparent that this strain contains many virulence factors (>40 hits against the VFDB and 25 hits against the Victors database [62,63]), including a CLpP protease homologue that has been shown to be involved in the regulation of virulence and stress responses in *Staphylococcus aureus*. This observation, together with the fact that both of the closest relatives, *Psd. oleovorans* and *Psd. mendocina,* are opportunistic human pathogens that have been found to cause bacteremia, endocarditis, and meningitis, indicates that this new *Pseudomonas* strain is potentially an opportunistic pathogen and should be characterized further. *Pseudomonas* sp. NSM was isolated from the location 1 pond during the March sampling but was found to present the most abundant in the July 2025 metagenomic datasets.

The presence of the pathogen *Vibrio cholerae* in the metagenomic analysis and its isolation from the October samples (Table 1) is intriguing. We previously analyzed the genome of this isolated species, compared it to other *Vibrio* species, and verified that this is indeed a species that belongs to the pathogenic *Vibrio cholerae* strains [64]. We isolated *Vibrio cholerae* from both locations by repetitive plating on aerobic grown plates. When plotting out the normalized relative abundance of *Vibrio cholerae* from the metagenomics data (Figure 6E,F), we noticed the highest presence in the October samples for location 1 and July for location 2. The genome of this new strain shows several potential virulence factors and antibiotic resistance markers [64], which illustrates that multidrug-resistant pathogenic *V. cholerae* can be found in remote environments like the Nebraska salt marshes.

A search of these new genomes in the CARD and PATRIC AMR databases showed that the genomes of *Rhodopseudomonas*, *Rheinheimera*, *Vibrio*, and *Pseudomonas*, all contain several multidrug efflux transporters, beta-lactamases, spectinomycin resistance, and macrolide specific efflux pumps. Noteworthy is also that the *Pseudomonas* genome contains at least three markers for resistance to fluoroquinolones, *Rheinheimera* contains clindamycin and pulvomycin resistance, and *Vibrio* contains a tetracycline resistance gene.

## 4. Discussion

The Nebraska salt marsh areas are a relic of ancient oceans that once covered central North America. Conservation of natural areas has historically typically emphasized plant and insect ecology; however, it is important to start at the base, by looking at the microbial changes that often show signs of distress or start recovery months or years before changes become evident in the larger ecosystem. It is therefore important to study and attempt to preserve these locally important ecosystems. To provide a better understanding of the Nebraska salt marsh micro-ecosystem, we analyzed and compared the microbiome throughout different seasons.

The overall microbiome appears to be susceptible to seasonal variations at each of the monitored locations; however, it also shows resilience and consistency over the two-year study period. The microbiome shows the highest diversity in the fall season (October sampling), which coincides with both locations showing the least water in the ponds and the highest salinity levels measured throughout the year. When studying the overall composition and the most abundant species in the various seasonal samples, we noticed a low abundance of cyanobacteria and eukaryotic algae. Although they were present in all samples, they were not among the most abundant species, with the exception of the July samples in location 1, where *Pseudanabaenales* and *Synechoccus* were found as being among the top taxa. Given the low water availability and high salinity in some of the drier seasons, halophilic bacteria and archaea are expected to be predominant; however, cyanobacteria and algae are commonly found in marine or coastal salt marshes in higher abundance, where they enrich the bacterial ecosystem with organic matter and nutrients through oxygenic photosynthesis [65,66]. In contrast, we found bacteria mainly belonging to *Rhodobacterales* to be consistently the most abundant overall in the seasonal samples (Figure 1 and Figure 4). *Rhodobacterales* contains mainly photosynthetic bacteria but also includes chemoheterotrophic species. The most abundant genera belonging to *Rhodobacterales* in our samples were *Yoonia* and *Rhodobacter*, which are both anoxygenic photoheterotrophs.

The genus *Yoonia* is known to be prevalent in high-altitude, cold, and less disturbed saline lake waters. Although the exact mechanisms are still under investigation, it has been suggested that it plays a significant role in the nitrogen cycle of these specific environments [67]. The abundance of *Yoonia*, especially in the colder months (March, October and December) in the Nebraska salt marshes appears to match well with its usual cold saline lake water habitat.

*Rhodobacter* is a genus of purple non-sulfur bacteria that grows photoheterotrophic and is facultatively anaerobic [68]. Purple non-sulfur bacteria play vital roles in various environmental processes, primarily through their diverse metabolic capabilities. They act as key players in nutrient cycling, particularly carbon and nitrogen, and are involved in detoxifying harmful substances like hydrogen sulfide. They are present in many aqueous and soil environments and have also been found in weakly and moderately mineralized soda lakes; however, only three truly alkaliphilic species are known [69,70,71]. Purple bacteria are able to photoautotrophically fix carbon or to consume it photoheterotrophically. Many non-sulfur purple bacteria are able to fix nitrogen, which is likely an important role they play in the Nebraska salt marshes.

The higher salinity samples (collected in October) showed a higher diversity of species but also included some more halophilic species. For example, at location 1, we noticed a high level of *Pontimonas*. This genus currently includes only one described and validly published species, *Pontimonas salivibrio* [72]. This species was isolated from a solar saltern, indicating a preference for saline environments and is photoheterotrophic. While at location 2, the more saline October samples included a higher abundance of *Halomonas*, which consists of halophilic or halotolerant species that have been isolated from marine, saline, or hypersaline environments and other saline habitats [73]. The LDA analysis also indicated that the major differentiating species between the seasonal samplings were all marine or marine or halophilic species, and the majority were photosynthetic species (Figure 5).

A reason for the low abundance of algae in the Nebraska salt marshes is likely that algae are typically limited by cold and low light: during colder seasons, lower water temperatures and reduced sunlight generally inhibit algae growth. This will lead to decreased metabolic rates and reproduction, resulting in lower algal populations. On the other hand, anoxygenic phototrophic bacteria can be more active in cold environments. There are many examples of extremophilic types found in extreme environments like polar regions and deep oceans that are capable of adapting to and thriving in cold conditions. Their cold adaptations include mechanisms like maintaining membrane fluidity and producing antifreeze proteins. There are some examples of cold-adapted green algae species [74,75]; however, these are typically not found in environments where they would be easily outcompeted by anoxygenic phototrophic bacteria.

Anoxygenic phototrophic bacteria do contribute to nutrient cycling even in cold and saline environments. Many species play vital roles in nutrient cycling (e.g., nitrogen, sulfur, etc.) in cold aquatic ecosystems. To this point, we extensively showed the presence of several phototrophs and chemotrophs that contribute to the sulfur cycle in the Nebraska salt marshes in our earlier report [15]. Our present LDA analysis also showed that many of the differentiating bacterial species were purple sulfur or non-sulfur bacteria. Their presence is consistent with our previous analysis of the sulfur redox cycling in the Nebraska salt marshes, which involves both phototrophic and chemotrophic bacteria (for a detailed overview of the sulfur cycling mechanism in the marshes, see [15]). In addition, several of the newly identified purple bacteria in our current report (e.g., *Rhodobacter* and *Rhodopseudomonas*) belong to families that are known to contribute to nitrogen cycling. The fact that the nitrate/nitrite levels were at relatively low levels for all seasons indicates that there is a balance in the nitrogen cycle maintained through the seasons in these marshes, and the high abundance of purple non-sulfur bacteria undoubtedly contribute to maintaining this balance. Anoxygenic phototrophic bacteria can also contribute to carbon cycling by breaking down organic matter, which facilitates the availability of nutrients for other organisms.

Given the abundance of purple bacteria in the metagenomic samples (and our cultivation conditions on RCVB plates), it is not surprising that most of the isolated and sequenced species belong to this versatile group of bacteria. Based on whole genome ANI comparisons, seven of the ten isolated strains appear to belong to potentially new species, and at least one may even belong to novel genus (*Roseinatronobacter* sp. NSM). Further physiological and morphological studies are underway to fully characterize these new isolates. Without further individual characterization of these isolated species, it is challenging to pinpoint their specific environmental roles in this ecosystem; however, based on their homology (and initial growth studies), it is clear that the isolated *Rhodobacterales* and *Hyphomicrobiales* species are well-adapted to the higher pH and salinity environments and have important roles in nitrogen cycling (several of the species have nitrogen-fixing homologues), Fe-S, and carbon cycling in general.

Overall, the purple non-sulfur species appear to be present in high relative abundance in all seasons, with the highest levels in the fall and spring (Figure 4, Figure 5 and Figure 6). Purple photosynthetic bacteria are known to exhibit seasonal variations in their physiology and community composition in response to environmental factors like light, temperature, and sulfide concentration. The spring and early fall season in Nebraska have an abundance of sunlight and suitable temperatures for these species to flourish. During the warmer summer months (July), there seems to be an increase in cyanobacterial species (e.g., *Anabeanopsis*, *Pseudanabaenales*, and *Synechococcales*), which also corresponds to the lower pH and salinity that we observed in July (Figure 1). These conditions are more suitable for these oxygenic phototrophic species to grow in higher abundance. Together with the increased plant growth, this provides an increased fixed carbon pool that will benefit decomposing organisms and microbial growth, especially in other seasons.

Anaerobic purple sulfur bacteria, *Reinheimera* and *Allochromatium*, seem to be most abundant and increasing in the fall (and winter) samples. These sampling times also have the higher pH and salinity (Figure 1) and less active plant and algal growth, which provides suitable anoxic conditions for these species to grow. These are known to be sulfur oxidizing species that play important roles in generating oxidized sulfur components that can be used for plant and algal growth in the subsequent seasons.

Our initial seasonal variation microbiome analysis mainly focused on photosynthetic organisms and their important roles in the main nutrient cycling (C, N, S), because these photosynthetic species were found to be the majority of high relative abundance species. However, there are certainly many chemotrophic species that also play essential roles in nutrient cycling. A few of these were also found to be significant differentiating factors in the seasonal LDA comparison, such as *Aquiflexum balticum*, *Muricauda ruestringensis*, and *Martelella mediterranea* species. In particular, *Aquiflexum balticum* has been found to be involved in the degradation of plankton blooms in an aquatic environment [43]. In addition, rhizobial species such as *Martelella mediterranea* play important roles in nutrient provision through nitrogen fixation and phosphate solubilization, plant growth promotion via plant hormone production, and stress tolerance by helping plants cope with drought, salinity, and heavy metals [76,77].

One surprising isolate was *Vibrio cholerae*, which is a known human intestinal pathogen [78,79]. Although *Vibrio cholerae* is found in many environments, including seawater and coastal aquatic areas, the high pH and saline Nebraska salt marshes are relatively isolated smaller bodies of water that are fed by groundwater and natural precipitation. This begs the question of what explains the seasonal occurrence of this species in relatively high amounts in the summer and fall seasons. Other studies of *Vibrio* outbreaks in coastal areas have also shown seasonal variations and similarly show increased activity in the fall seasons, which is likely due to optimal temperatures, pH, dissolved oxygen, and salinity levels that can contribute to these fluctuations [80,81]. In addition, a few studies have investigated the potential of the airborne transmission of pathogens (with a focus on *Vibrio* and *Pseudomonas*) from and to seawater and coastal areas [82,83,84]. It is intriguing to speculate about the possible environmental factors or potential animal vectors that could contribute to the presence of *Vibrio*, *Pseudomonas*, and other pathogens in these isolated bodies of water and their seasonal variations. Future studies should look at airborne vectors or bird migration patterns that could bring in or transfer these pathogens from and to nearby neighborhoods. With increasing urban development near the Nebraska salt marshes there are increasing challenges with effluent contaminating the marshes or, alternatively, potential transfer of these pathogenic and AMR containing species to neighboring developments.

The increase in environmental AMR markers is of growing global concern [85,86,87,88] and has impacts not only on the environmental microbiomes but also for healthcare and disease management. We found several potential AMR markers in our metagenomic datasets that show seasonal variations. The highest levels of AMR genes were found during the spring (March) and fall (October), although some markers were consistently present during all seasons, for example clindamycin resistance. In addition, in our isolated species genomes, we identified AMR genes in several of the genomes. Of particular interest is the presence of tetracycline resistance in *Vibrio cholerae* (Tetracycline resistance MFS efflux pump Tet(35)) [89]. Tetracyclines have long been the antibiotics of choice for treating severe cholera effectively worldwide. However, tetracycline-resistant strains of *V. cholerae* are being increasingly reported worldwide [90,91]. Although these resistant strains have been responsible for major epidemics in Latin America, Asia, and Africa, the widespread presence of tetracycline resistance in *Vibrio cholerae* is still understudied [84,92]. This makes the presence of TetR and other multiple resistance genes in *Vibrio* and the other new isolates from these saline and alkaline environments even more relevant.

Further cultivation studies are underway to test the antibiotic susceptibility range on these species. Nevertheless, there seems to be a relatively high abundance and variety of AMR markers present in the Nebraska salt marshes, and finding these in the somewhat isolated areas with little anthropogenic influence is a concern. Similar to the discussion on pathogen transmission, one should be concerned with the source of these AMR genes and the potential transmission to other nearby ecosystems and neighborhoods. As mentioned above, there has been an increase in urban and agricultural development near the Nebraska salt marshes in Lincoln in the past decades, which possibly contributed to the transmission issues of AMR genes through effluent contamination. Further impact studies will possibly provide more clarity on these issues.

In conclusion, throughout the year the salt marsh microbiome appears to be dominated by anoxygenic phototrophs, of which many belong to both purple non-sulfur and purple sulfur bacterial species. Of the few that we isolated and tested, the majority appear to be novel species based on their genomic analysis. This indicates the importance of these phototrophs in the salt marsh habitat. They obtain most of their energy from photosynthesis and are responsible for nutrient cycling, including carbon, nitrogen, and sulfur cycling in this natural environment, thereby providing accessible nutrients for plant and insect growth in the salt marsh habitat. Some of this role is also performed by oxygenic phototrophs like cyanobacteria and eukaryotic algae (as in many aquatic habitats around the world); however, these are only present in significant abundance during the summer months. The higher abundance of anoxygenic species throughout the year could result in low oxidation levels in the ponds outside the summer months, which provides opportunities for facultative anaerobes like purple non-sulfur bacteria to flourish.

In 2003, the Nature Conservancy helped form the Saline Wetlands Conservation Partnership to start preserving the last remnants of Nebraska’s saline wetlands. The Marsh Wren saline wetland restoration project was started to conserve and restore approximately 150 acres saline wetlands and other habitats of the lower Salt Creek valley and was completed in 2017 [20]. This restoration used a combination of traditional restoration methods and the physical manipulation of hydrology through the pumping of saline groundwater to the wetland surface. Since then, there have been ongoing efforts to study the restoration and preservation of the native plant and insect community in these salt marshes; however, little attention has been given to the microbiome and its impact on the preservation of this native habitat. Certainly, understanding and maintaining a healthy environmental microbiome is more complex and challenging as compared to the more tangible plant and insect community. Nevertheless, it has been shown in many studies that a well-adapted and healthy microbiome is essential to achieve the sustainable restoration of native areas [59,93,94,95,96,97]. Ultimately, the microbiome is needed for nutrient cycling and upscaling for plant and insect bioavailability.

Now that we have a better understanding of the temporal and spatial variations and resilience of the Nebraska salt marsh microbiome, future restoration plans should include prebiotic and probiotic considerations to maintain these important microorganisms that are well adapted to these unique conditions. With a growing global loss of biodiversity, there is a need for testing and implementing probiotic and prebiotic strategies [80], and the isolated nature of the Nebraska salt marshes makes an ideal scenario for implementing and monitoring microbial stewardship plans. It has been proposed, in general, that plans for microbiome management can include direct supplementation of defined microbes or consortia with beneficial properties (provided they are known) or adding microbiota-active metabolites (such as prebiotics) to an ecosystem (or host) or a mixture of probiotic and prebiotic solutions (synbiotics) [94,98]. Another facet of microbial stewardship is also reducing the ecosystem (or host) exposure to factors such as antimicrobial toxins or pollutants. For example, in the case of our environmental ecosystem, this includes future monitoring and preventing the spread of pathogenic organisms and antibiotic resistance genes from and to these salt marsh areas. The implementation of such pre- and probiotic plans has been hampered by a lack of basic understanding of the composition of the microbiome and the ecological mechanisms that drive the temporal fluctuations in environmental microbiomes. Establishing baselines with seasonal and spatial variations, as we presented here, are a necessary starting point for future microbial stewardship plans. A comparison of the Nebraska salt marsh microbiome to other coastal or inland salt marsh microbiomes could also provide common strategies that could help preserve and restore other endangered salt marsh ecosystems.

## Figures and Tables

**Table 1:** Overview of the isolated bacterial species from the Nebraska salt marsh locations and their whole genome characteristics.

Species	Size	% GC	Coverage	Contigs	N50	CDS	tRNAs	Closest relative ^1^	ANI % ^2^	Accession	Family	Order
***Rhodobacter* sp. *NSM***	4.6 Mb	67.7	189x	81	384,670	4658	48	*Rhodobacter megalophilus* DSM 18937	84.4	JBQPXH000000000	*Rhodobacteraceae*	*Rhodobacterales*
***Roseinatronobacter* sp. *NSM***	4.1 Mb	60.3	93x	108	93,431	4196	42	*Roseinatronobacter thiooxidans* DSM 13087	75.8	JBQTCF000000000	*Rhodobacteraceae*	*Rhodobacterales*
***Paracoccus* sp. *NSM***	3.5 MB	67.3	125x	40	316,482	3267	41	*Paracoccus bogoriensis* BOG6	89	JBQTCG000000000	*Paracoccaceae*	*Rhodobacterales*
***Rhodopseudomonas* sp., *NSM***	5.3 Mb	66	182x	21	416,869	4772	51	*Rhodopseudomonas* sp. HaA2	90.1	JBQTCH000000000	*Nitrobacteraceae*	*Hyphomicrobiales*
***Salinarimonas* sp. *NSM***	4.7 Mb	71.8	114x	133	55,398	4692	43	*Salinarimonas rosea* DSM 21201	88.9	JBQTCJ000000000	*Salinarimonadaceae*	*Hyphomicrobiales*
***Marinobacter* sp. *NSM***	3.8 Mb	57.3	107x	22	602,037	3595	46	*Marinobacter* sp. THAF39	94.8	JBQTCK000000000	*Phyllobacteriaceae*	*Hyphomicrobiales*
***Rheinheimera* sp. *NSM***	4.3 Mb	50.8	125x	21	617,608	4029	60	*Rheinheimera* sp. YQF-2	84.5	JBQTCI000000000	*Chromatiaceae*	*Chromatiales*
***Oceanimonas smirnovii NSM***	3.3 Mb	56	100x	58	233,274	3233	66	*Oceanimonas smirnovii* ATCC BAA-899	98.1	JBQRTU000000000	*Aeromonadaceae*	*Aeromonadales*
***Ectopseudomonas oleovorans NSM***	5.3 Mb	64.9	54x	69	265,885	5038	66	*Ectopseudomonas oleovorans* ZKA50	98.76	JBQTDA000000000	*Pseudomonadaceae*	*Pseudomonadales*
***Vibrio cholerae NSM***	4.2 Mb	47.4	46x	99	259,308	3779	84	*Vibrio cholerae* RFB16	98	JBKFFD000000000	*Vibrionaceae*	*Vibrionales*

^1^ Closest genome relative was deteremined using the ‘Similar genome finder’ function in BV-BRC; ^2^ ANI is to the closest relative genome, calculated using Jspecies.

## Data Availability

The WGS metagenomic sequencing datasets and the WGS datasets of the purified isolates were deposited into the NCBI Genbank under BioProject PRJNA1308161. The metagenomic datasets are accessible with the following SRA numbers: SRR35078099 through SRR35078121. The WGS sequences of the isolates were submitted under the same BioProject with the following accession numbers: JBQPXH000000000; JBQTCF000000000; JBQTCG000000000; JBQTCH000000000; JBQTCJ000000000; JBQTCK000000000; JBQTCI000000000; JBQRTU000000000; JBQTDA000000000. The *Vibrio cholerae* NSM genome sequence was deposited under a different BioProject PRJNA1202057, with the accession JBKFFD000000000. The version described in this paper is version JBKFFD010000000. The raw sequencing reads have been submitted to SRA, and the corresponding accession number is SRR31807841.

## Supplementary Materials

The following supporting information can be downloaded at https://www.mdpi.com/article/10.3390/microorganisms13102369/s1: Table S1: Results of the alpha-diversity for each sample calculated as the Shannon diversity, Simpson diversity, Simpson reciprocal index, Berger–Parker index, and Fisher’s index; Table S2: Results of the Metagenomic Read Mapping against the CARD database performed in BV-BRC for each of the samples. A total of 21 AMR targets were identified in the samples.

## Abbreviations

The following abbreviations are used in this manuscript:

AMR	Antimicrobial resistance
ANI	Average nucleotide identity
LDA	Linear discriminant analysis
PCA	Principal component analysis
PNS	Purple non-sulfur bacteria
WGS	Whole genome sequence

## References

[1] Bhowmik S. (2022). Ecological and economic importance of wetlands and their vulnerability. Research Anthology on Ecosystem Conservation and Preserving Biodiversity.

[2] Xin P., Wilson A., Shen C., Ge Z., Moffett K.B., Santos I.R., Chen X., Xu X., Yau Y.Y.Y., Moore W. (2022). Surface water and groundwater interactions in salt marshes and their impact on plant ecology and coastal biogeochemistry. Rev. Geophys..

[3] Bertness M., Silliman B. (2004). Salt Marshes under siege. Am. Sci..

[4] Campbell A.D., Fatoyinbo L., Goldberg L., Lagomasino D. (2022). Global hotspots of salt marsh change and carbon emissions. Nature.

[5] Álvarez-Rogel J., Jiménez-Cárceles F.J., Roca M.J., Ortiz R. (2007). Changes in soils and vegetation in a Mediterranean coastal salt marsh impacted by human activities. Estuar. Coast. Shelf Sci..

[6] Schuerch M., Kiesel J., Boutron O., Guelmami A., Wolff C., Cramer W., Caiola N., Ibáñez C., Vafeidis A.T. (2025). Large-scale loss of Mediterranean coastal marshes under rising sea levels by 2100. Commun. Earth Environ..

[7] https://marine-conservation.org/on-the-tide/mediterranean-mpas-falling-short.

[8] Joeckel R.M., Clement B.A. (1999). Surface features of the Salt Basin of Lancaster County, Nebraska. CATENA.

[9] Johnsgard P.A. (2001). The Nature of Nebraska: Ecology and Biodiversity.

[10] Ungar I., Hogan W., McClelland M. (1969). Plant communities of saline soils at Lincoln, Nebraska. Am. Midl. Nat..

[11] Panella M. (2012). Nebraska’s At-Risk Species Wildlife.

[12] Brosius T.R., Higley L.G. (2013). Behavioral niche partitioning in a sympatric tiger beetle assemblage and implications for the endangered Salt Creek tiger beetle. PeerJ.

[13] Gibbens S. (2020). The remnants of a vast prehistoric sea lie hidden in Nebraska’s endangered marshes. National Geographic.

[14] Gao J., Liu M., Shi S., Liu Y., Duan Y., Lv X., Bohu T., Li Y., Hu Y., Wang N. (2021). Disentangling responses of the subsurface microbiome to wetland status and implications for indicating ecosystem functions. Microorganisms.

[15] Athen S.R., Dubey S., Kyndt J.A. (2021). The Eastern Nebraska Salt Marsh Microbiome Is Well Adapted to an Alkaline and Extreme Saline Environment. Life.

[16] Bodelier P.L., Dedysh S.N. (2013). Microbiology of wetlands. Front. Microbiol..

[17] Lovell C.R., Davis D.A. (2012). Specificity of Salt Marsh Diazotrophs for Vegetation Zones and Plant Hosts: Results from a North American marsh. Front. Microbiol..

[18] Liang S., Li H., Wu H., Yan B., Song A. (2023). Microorganisms in coastal wetland sediments: A review on microbial community structure, functional gene, and environmental potential. Front. Microbiol..

[19] Núñez A., García A.M., Moreno D.A., Guantes R. (2021). Seasonal changes dominate long-term variability of the urban air microbiome across space and time. Environ. Int..

[20] Malmstrom T. (2019). Saline Wetlands Conservation Partnership 2018 Progress Report.

[21] Fiore N.A., Dunigan D.D., Shaffer J.J., Roberts R., Antony-Babu S., Plantz B.A., Nickerson K.W., Benson A.K., Weber K.A. (2019). Microbial Community of Saline, Alkaline Lakes in the Nebraska Sandhills Based on 16S rRNA Gene Amplicon Sequence Data. Microbiol. Resour. Announc..

[22] Pfennig N. (1975). The phototrophic bacteria and their role in the sulfur cycle. Plant Soil.

[23] Wattam A.R., Davis J.J., Assaf R., Boisvert S., Brettin T., Bun C., Conrad N., Dietrich E.M., Disz T., Gabbard J.L. (2017). Improvements to PATRIC, the all-bacterial Bioinformatics Database and Analysis Resource Center. Nucleic Acids Res..

[24] Lu J., Rincon N., Wood D.E., Breitwieser F.P., Pockrandt C., Langmead B., Salzberg S.L., Steinegger M. (2022). Metagenome analysis using the Kraken Software Suite. Nat. Protoc..

[25] Wood D.E., Lu J., Langmead B. (2019). Improved metagenomic analysis with Kraken 2. Genome Biol..

[26] Metsalu T., Vilo J. (2015). ClustVis: A web tool for visualizing clustering of multivariate data using Principal Component Analysis and heatmap. Nucleic Acids Res..

[27] Anderson M.J., Ellingsen K.E., McArdle B.H. (2006). Multivariate dispersion as a measure of beta diversity. Ecol. Lett..

[28] Bray J.R., Curtis J.T. (1957). An Ordination of the Upland Forest Communities of Southern Wisconsin. Ecol. Monogr..

[29] Segata N., Izard J., Walron L., Gevers D., Miropolsky L., Garrett W., Huttenhower C. (2011). Metagenomic Biomarker Discovery and Explanation. Genome Biol..

[30] Clausen P.T., Aarestrup F.M., Lund O. (2018). Rapid and precise alignment of raw reads against redundant databases with KMA. BMC Bioinform..

[31] Alcock B.P., Raphenya A.R., Lau T.T.Y., Tsang K.K., Bouchard M., Edalatmand A., Huynh W., Nguyen A.-L.V., Cheng A.A., Liu S. (2020). CARD 2020: Antibiotic resistome surveillance with the comprehensive antibiotic resistance database. Nucleic Acids Res..

[32] Wick R.R., Judd L.M., Gorrie C.L., Holt K.E. (2017). Unicycler: Resolving bacterial genome assemblies from short and long sequencing reads. PLoS Comput. Biol..

[33] Parks D.H., Imelfort M., Skennerton C.T., Hugenholtz P., Tyson G.W. (2014). CheckM: Assessing the quality of microbial genomes recovered from isolates, single cells, and metagenomes. Genome Res..

[34] Aziz R., Bartels D., Best A., DeJongh M., Disz T., Edwards R., Formsma K., Gerdes S., Glass E.M., Kubal M. (2008). The RAST server: Rapid annotations using subsystems technology. BMC Genom..

[35] Ondov B.D., Treangen T.J., Melsted P., Mallonee A.B., Bergman N.H., Koren S., Phillippy A.M. (2016). Mash: Fast genome and metagenome distance estimation using MinHash. Genome Biol..

[36] Richter M., Rosselló-Móra R., Glöckner F.O., Peplies J. (2016). JSpeciesWS: A web server for prokaryotic species circumscription based on pairwise genome comparison. Bioinformatics.

[37] Imhoff J.F., Kyndt J.A., Meyer T.E. (2022). Genomic Comparison, Phylogeny and Taxonomic Reevaluation of the *Ectothiorhodospiraceae* and Description of *Halorhodospiraceae* fam. nov. and Halochlorospira gen. nov. Microorganisms.

[38] Weissgerber T., Dobler N., Polen T., Latus J., Stockdreher Y., Dahl C. (2013). Genome-Wide Transcriptional Profiling of the Purple Sulfur Bacterium Allochromatium vinosum DSM 180T during Growth on Different Reduced Sulfur Compounds. J. Bacteriol..

[39] Yurkov V.V., Beatty J.T. (1998). Aerobic Anoxygenic Phototrophic Bacteria. Microbiol. Mol. Biol. Rev..

[40] Kyndt J.A., Robertson S., Shoffstall I.B., Ramaley R.F., Meyer T.E. (2022). Genome Sequence and Characterization of a Xanthorhodopsin-Containing, Aerobic Anoxygenic Phototrophic *Rhodobacter* Species, Isolated from Mesophilic Conditions at Yellowstone National Park. Microorganisms.

[41] Winckelmann D., Bleeke F., Bergmann P., Klöck G. (2015). Growth of *Cyanobacterium aponinum* influenced by increasing salt concentrations and temperature. 3 Biotech.

[42] Lin J.-Y., Ng I.-S. (2023). Thermal cultivation of halophilic *Cyanobacterium aponinum* for C-phycocyanin production and simultaneously reducing carbon emission using wastewater. Chem. Eng. J..

[43] Brettar I., Christen R., Höfle M.G. (2004). *Aquiflexum balticum* gen. nov., sp. nov., a novel marine bacterium of the *Cytophaga*-*Flavobacterium*-*Bacteroides* group isolated from surface water of the central Baltic Sea. Int. J. Syst. Evol. Microbiol..

[44] Hameed A., Shahina M., Lin S.Y., Sridhar K.R., Young L.S., Lee M.R., Chen W.M., Chou J.H., Young C.C. (2012). *Siansivirga zeaxanthinifaciens* gen. nov., sp. nov., a novel zeaxanthin-producing member of the family Flavobacteriaceae isolated from coastal seawater of Taiwan. FEMS Microbiol Lett..

[45] Bruns A., Rohde M., Berthe-Corti L. (2001). *Muricauda ruestringensis* gen. nov., sp. nov., a facultatively anaerobic, appendaged bacterium from German North Sea intertidal sediment. Int. J. Syst. Evol. Microbiol..

[46] Rivas R. (2005). *Martelella mediterranea* gen. nov., sp. nov., a novel -proteobacterium isolated from a subterranean saline lake. Int. J. Syst. Evol. Microbiol..

[47] McArthur A.G., Waglechner N., Nizam F., Yan A., Azad M.A., Baylay A.J., Bhullar K., Canova M.J., De Pascale G., Ejim L. (2013). The comprehensive antibiotic resistance database. Antimicrob. Agents Chemother..

[48] Lapthorne S., McWade R., Scanlon N., Ní Bhaoill S., Page A., O’Donnell C., Dornikova G., Hannan M., Lynch B., Lynch M. (2024). Rising clindamycin resistance in group A Streptococcus in an Irish healthcare institution. Access Microbiol..

[49] Heß S., Gallert C. (2014). Resistance behaviour of inducible clindamycin-resistant staphylococci from clinical samples and aquatic environments. J. Med. Microbiol..

[50] Samreen I.A., Hesham A.M., Hussein H.A. (2021). Environmental antimicrobial resistance and its drivers: A potential threat to public health. J. Glob. Antimicrob. Resist..

[51] Assefa M. (2022). Inducible Clindamycin-Resistant *Staphylococcus aureus* Strains in Africa: A Systematic Review. Int. J. Microbiol..

[52] Arunasri K., Venkata Ramana V., Spröer C., Sasikala C., Ramana C.V. (2008). *Rhodobacter megalophilus* sp. nov., a phototroph from the Indian Himalayas possessing a wide temperature range for growth. Int. J. Syst. Evol. Microbiol..

[53] Koh R.-H., Song H.-G. (2007). Effects of application of *Rhodopseudomonas* sp. on seed germination and growth of tomato under axenic conditions. J. Microbiol. Biotechnol..

[54] Xu J., Feng Y., Wang Y., Lin X. (2018). Effect of Rhizobacterium *Rhodopseudomonas palustris* Inoculation on *Stevia rebaudiana* Plant Growth and Soil Microbial Community. Pedosphere.

[55] Hsu S.-H., Shen M.-W., Chen J.-C., Lur H.-S., Liu C.-T. (2021). The Photosynthetic Bacterium *Rhodopseudomonas palustris* Strain PS3 Exerts Plant Growth-Promoting Effects by Stimulating Nitrogen Uptake and Elevating Auxin Levels in Expanding Leaves. Front. Plant Sci..

[56] Batool K., Tuz Zahra F., Rehman Y. (2017). Arsenic-Redox Transformation and Plant Growth Promotion by Purple Nonsulfur Bacteria *Rhodopseudomonas palustris* CS2 and *Rhodopseudomonas faecalis* SS5. BioMed Res. Int..

[57] Kantha T., Kantachote D., Klongdee N. (2015). Potential of biofertilizers from selected *Rhodopseudomonas palustris* strains to assist rice (*Oryza sativa* L. subsp. indica) growth under salt stress and to reduce greenhouse gas emissions. Ann. Microbiol..

[58] Kantachote D., Nunkaew T., Kantha T., Chaiprapat S. (2016). Biofertilizers from *Rhodopseudomonas palustris* strains to enhance rice yields and reduce methane emissions. Appl. Soil Ecol..

[59] Hobbs A., Ochoa-Rojas D., Humphrey C.E., Kyndt J.A., Moore T.C. (2025). Soil microbiome perturbation impedes growth of *Bouteloua curtipendula* and increases relative abundance of soil microbial pathogens. bioRxiv.

[60] Oda Y., Larimer F.W., Chain P.S., Malfatti S., Shin M.V., Vergez L.M., Hauser L., Land M.L., Braatsch S., Beatty J.T. (2008). Multiple genome sequences reveal adaptations of a phototrophic bacterium to sediment microenvironments. Proc. Natl. Acad. Sci. USA.

[61] Imhoff J.F., Meyer T.E., Kyndt J. (2020). Genomic and genetic sequence information of strains assigned to the genus *Rhodopseudomonas* reveal the great heterogeneity of the group and identify strain *Rhodopseudomonas palustris* DSM 123T as the authentic type strain of this species. Int. J. Syst. Evol. Microbiol..

[62] Mao C., Abraham D., Wattam A.R., Wilson M.J., Shukla M., Yoo H.S., Sobral B.W. (2015). Curation, integration and visualization of bacterial virulence factors in PATRIC. Bioinformatics.

[63] Chen L., Zheng D., Liu B., Yang J., Jin Q. (2016). VFDB 2016: Hierarchical and refined dataset for big data analysis-10 years on. Nucleic Acids Res..

[64] Kyndt J.A. (2025). *Vibrio cholerae* genome isolated from the Nebraska salt marshes contains several antibiotic resistance markers. Microbiol. Resour. Announc..

[65] Stal L.J., Gemerden H., Krumbein W.E. (1985). Structure and development of a benthic marine microbial mat. FEMS Microbiol. Lett..

[66] Bolhuis H., Stal L.J. (2011). Analysis of bacterial and archaeal diversity in coastal microbial mats using massive parallel 16S rRNA gene tag sequencing. ISME J..

[67] Zhao Z., Zhao Y., Marotta F., Xamxidin M., Li H., Xu J., Hu B., Wu M. (2024). The microbial community structure and nitrogen cycle of high-altitude pristine saline lakes on the Qinghai-Tibetan plateau. Front. Microbiol..

[68] Imhoff J.F., Trujillo M.E., Dedysh S., DeVos P., Hedlund B., Kämpfer P., Rainey F.A., Whitman W.B. (2015). *Rhodobacter*. Bergey’s Manual of Systematics of Archaea and Bacteria.

[69] Boldareva E.N., Moskalenko A.A., Makhneva Z.K., Tourova T.P., Kolganova T.V., Gorlenko V.M. (2009). *Rubribacterium polymorphum* gen. nov., sp. nov., a novel alkaliphilic nonsulfur purple bacterium from an Eastern Siberian soda lake. Microbiology.

[70] Milford A.D., Achenbach L.A., Jung D.O., Madigan M.T. (2000). *Rhodobaca bogoriensis* gen. nov. and sp. nov. alcaliphilic purple nonsulfur bacterium from African Rift valley soda lakes. Arch. Microbiol..

[71] Boldareva E.N., Akimov V.N., Boychenko V.A., Stadnichuk I.N., Moskalenko A.A., Makhneva Z.K., Gorlenko V.M. (2008). *Rhodobaca barguzinensis* sp. nov., a new alkaliphilic purple sulfur bacterium isolated from a soda lake of the Barguzin valley (Buryat Republic, Eastern Siberia). Microbiology.

[72] Jang G.I., Cho Y., Cho B.C. (2013). *Pontimonas salivibrio* gen. nov., sp. nov., a new member of the family *Microbacteriaceae* isolated from a seawater reservoir of a solar saltern. Int. J. Syst. Evol. Microbiol..

[73] Vreeland R.H., Whitman W.B. (2015). “Halomonas”. Bergey’s Manual of Systematics of Archaea and Bacteria.

[74] Peng Z., Liu G., Huang K. (2021). Cold Adaptation Mechanisms of a Snow Alga *Chlamydomonas nivalis* During Temperature Fluctuations. Front. Microbiol..

[75] Morales-Sánchez D., Schulze P.S.C., Kiron V., Wijffels R.H. (2020). Temperature-Dependent Lipid Accumulation in the Polar Marine Microalga *Chlamydomonas malina* RCC2488. Front. Plant Sci..

[76] Vejan P., Abdullah R., Khadiran T., Ismail S., Nasrulhaq Boyce A. (2016). Role of Plant Growth Promoting Rhizobacteria in Agricultural Sustainability—A Review. Molecules.

[77] Chieb M., Gachomo E.W. (2023). The role of plant growth promoting rhizobacteria in plant drought stress responses. BMC Plant Biol..

[78] Ali M., Nelson A.R., Lopez A.L., Sack D.A. (2015). Updated global burden of cholera in endemic countries. PLoS Neglected Trop. Dis..

[79] Montero D.A., Vidal R.M., Velasco J., George S., Lucero Y., Gómez L.A., Carreño L.J., García-Betancourt R., O’Ryan M. (2023). *Vibrio cholerae*, classification, pathogenesis, immune response, and trends in vaccine development. Front. Med..

[80] Grimes D.J. (1991). Ecology of estuarine bacteria capable of causing human disease: A review. Estuaries.

[81] Brumfield K.D., Chen A.J., Gangwar M., Usmani M., Hasan N.A., Jutla A.S., Huq A., Colwell R.R. (2023). Environmental Factors Influencing Occurrence of *Vibrio parahaemolyticus* and *Vibrio vulnificus*. Appl. Environ. Microbiol..

[82] Shen S., Wu W., Grimes D.J., Saillant E.A., Griffitt R.J. (2020). Community composition and antibiotic resistance of bacteria in bottlenose dolphins *Tursiops truncatus*—Potential impact of 2010 BP Oil Spill. Sci. Total Environ..

[83] Pendergraft M.A., Grimes D.J., Giddings S.N., Feddersen F., Beall C.M., Lee C., Santander M.V., Prather K.A. (2021). Airborne transmission pathway for coastal water pollution. PeerJ.

[84] Rouard C., Collet L., Njamkepo E., Jenkins C., Sacheli R., Benoit-Cattin T., Figoni J., Weill F.X. (2024). Long-Distance Spread of a Highly Drug-Resistant Epidemic Cholera Strain. N. Engl. J. Med..

[85] Larsson D.G.J., Flach C.F. (2022). Antibiotic resistance in the environment. Nat. Rev. Microbiol..

[86] Tang K.W.K., Millar B.C., Moore J.E. (2023). Antimicrobial Resistance (AMR). Br. J. Biomed. Sci..

[87] Ifedinezi O.V., Nnaji N.D., Anumudu C.K., Ekwueme C.T., Uhegwu C.C., Ihenetu F.C., Obioha P., Simon B.O., Ezechukwu P.S., Onyeaka H. (2024). Environmental Antimicrobial Resistance: Implications for Food Safety and Public Health. Antibiotics.

[88] Saxena D., Gwalani R., Yadav A., Shah R. (2025). Growing Concerns on Antimicrobial Resistance—Past, Present, and Future Trends. Indian J. Community Med..

[89] Teo J.W., Tan T.M., Poh C.L. (2002). Genetic determinants of tetracycline resistance in *Vibrio harveyi*. Antimicrob. Agents Chemother..

[90] Faruque A.S., Alam K., Malek M.A., Khan M.G., Ahmed S., Saha D., Khan W.A., Nair G.B., Salam M.A., Luby S.P. (2007). Emergence of multidrug-resistant strain of *Vibrio cholerae* O1 in Bangladesh and reversal of their susceptibility to tetracycline after two years. J. Health Popul. Nutr..

[91] Kar S.K., Pal B.B., Khuntia H.K., Achary K.G., Khuntia C.P. (2015). Emergence and spread of tetracycline resistant *Vibrio cholerae* O1 El Tor variant during 2010 cholera epidemic in the tribal areas of Odisha, India. Int. J. Infect. Dis..

[92] Ahmadi M.H. (2021). Global status of tetracycline resistance among clinical isolates of *Vibrio cholerae*: A systematic review and meta-analysis. Antimicrob. Resist. Infect. Control.

[93] Lynum C.A., Bulseco A.N., Dunphy C.M., Osborne S.M., Vineis J.H., Bowen J.L. (2020). Microbial community response to a passive salt marsh restoration. Estuaries Coasts.

[94] Iqbal S., Begum F., Nguchu B.A., Claver U.P., Shaw P. (2025). The invisible architects: Microbial communities and their transformative role in soil health and global climate changes. Environ. Microbiome.

[95] Wang X., Chi Y., Song S. (2024). Important soil microbiota’s effects on plants and soils: A comprehensive 30-year systematic literature review. Front. Microbiol..

[96] Corinaldesi C., Bianchelli S., Candela M., Dell’Anno A., Gambi C., Rastelli E., Varrella S., Danovaro R. (2023). Microbiome-assisted restoration of degraded marine habitats: A new nature-based solution?. Front. Mar. Sci..

[97] Farrer E.C., Van Bael S.A., Clay K., Smith M.K.H. (2022). Plant-Microbial Symbioses in Coastal Systems: Their Ecological Importance and Role in Coastal Restoration. Estuaries Coasts.

[98] Peixoto R.S., Voolstra C.R., Sweet M., Duarte C.M., Carvalho S., Villela H., Lunshof J.E., Gram L., Woodhams D.C., Walter J. (2022). Harnessing the microbiome to prevent global biodiversity loss. Nat. Microbiol..

